# Therapeutic Challenges in Case of *Trichophyton indotineae* Dermatophytosis, Singapore, 2025 

**DOI:** 10.3201/eid3206.251606

**Published:** 2026-06

**Authors:** Tiara Joy Foo, Matthew Chung Yi Koh, Ka Lip Chew, Lester Juay, Sean Jiawei Wu

**Affiliations:** National University Hospital, Singapore

**Keywords:** Trichophyton indotineae, fungi, dermatophytosis, antifungal resistance, antimicrobial resistance, terbinafine, itraconazole, fungal infections, echinocandins, anidulafungin, Singapore

## Abstract

*Trichophyton indotineae* is an emerging dermatophyte frequently associated with terbinafine resistance. We report a case of recalcitrant *T. indotineae* infection in Singapore with limited response despite prolonged azole therapy, which only resolved after combination therapy with anidulafungin and itraconazole. This case highlights therapeutic challenges and need for improved diagnostics in *T. indotineae* infections.

Dermatophytosis is a globally prevalent superficial fungal infection predominantly caused by *Trichophyton* species. *T. indotineae* has emerged globally and is frequently associated with terbinafine resistance, although susceptibility is not universal (*1*). Treatment options are limited and often require prolonged systemic therapy. We describe a case of recalcitrant dermatophytosis in Singapore and its management challenges; clinical resolution occurred only after combined treatment with anidulafungin and itraconazole. Informed consent was obtained from the patient for publication.

## The Study

A 68-year-old man with hypertension sought care at National University Hospital, Singapore, for a 3-year history of recurrent pruritic groin plaques. Tinea cruris had been diagnosed initially by his general practitioner, and he received multiple courses of topical (clotrimazole and miconazole) and oral (griseofulvin, terbinafine, and itraconazole capsules) antifungal drugs; improvement was transient despite adherence. He reported intermittent use of over-the-counter topical corticosteroid-containing creams. Over the course of 3 years, lesions extended to the abdomen and gluteal region.

Initial examination showed extensive, sharply marginated, annular plaques with raised, scaly borders and central clearing over the lower abdomen, inguinal folds, gluteal region, and inner thigh (Figure, panel A); we noted no nail or scalp involvement. He was afebrile with no lymphadenopathy or systemic findings. His hemoglobin A1c, renal, and liver function test results were unremarkable. A fourth-generation HIV assay was negative. He was not on immunosuppressive therapy. 

**Figure:**
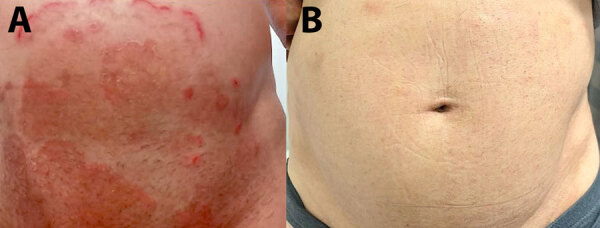
Skin lesions in case of *Trichophyton indotineae* dermatophytosis, Singapore, 2025. A) Before treatment; B) after 6 weeks of anidulafungin and itraconazole.

Skin scrapings were obtained for fungal culture. Matrix-assisted laser desorption/ionization time-of-flight mass spectrometry using the VITEK MS System Knowledge Base version 3.2 (bioMérieux, https://www.biomerieux.com) identified the isolate as *T. interdigitale*, likely because of the absence of *T. indotineae* in the reference database. Sanger sequencing of the internal transcribed spacer 1 region identified *T. indotineae*, which was thereafter established by whole-genome sequencing as well. At the time of management, clinical susceptibility testing was unavailable. 

The patient was started on itraconazole oral capsules at 200 mg/day. Itraconazole levels checked 1 week into treatment were below the assay’s limit of detection (<0.50 mg/L) despite adherence. Although target serum concentrations for dermatophytes are not well established, this finding raised concerns about inadequate bioavailability. The itraconazole dose was increased to 200 mg 2×/day. However, despite 6 weeks of therapy, the rash progressed. Adjunctive topical clotrimazole was applied to localized areas 2×/day, and strict hygiene measures were advised. We did not recheck itraconazole levels because further dose escalation was deemed unfeasible. After 6 months, itraconazole was stopped because of limited response.

Voriconazole was started after shared decision-making but discontinued after 3 days because of neuropsychiatric side effects. Combination therapy of intravenous anidulafungin (100 mg/d) and oral itraconazole (capsules 200 mg 2×/d) was selected empirically because of prolonged disease, partial response to azoles, and limited therapeutic options, not on the basis of in vitro synergy data. Off-label echinocandin use was undertaken after informed consent. This regimen finally resulted in marked flattening of plaques within 2 weeks. Treatment continued for 6 weeks through outpatient parenteral antimicrobial therapy; mild postinflammatory hyperpigmentation was noted at completion (Figure, panel B). The patient experienced no adverse effects, and liver function test results remained unremarkable. Follow-up fungal culture demonstrated no dermatophyte growth. The patient was in sustained remission at 6 months after therapy.

*T. indotineae* is an emerging cause of refractory dermatophytosis and causes considerable illness (*1*). The fungus is transmitted through direct contact or fomites, and manifests as pruritic, scaly, annular plaques. Terbinafine resistance is commonly linked to squalene epoxidase gene mutations (*1*,*2*), leading to elevated terbinafine MICs and clinical failure. Because clinical breakpoints are not established, MICs are often interpreted using European Committee on Antimicrobial Susceptibility Testing dermatophyte methodology and wild-type upper limits or epidemiological cutoff value–based approaches (*3*). A study from France proposed terbinafine MIC >0.2 mg/L as a threshold associated with resistance (*4*). In a series from the United Kingdom, 75% of isolates exhibited elevated terbinafine MICs (>0.5 mg/L) (*5*). However, clinical failure has been reported even with low terbinafine MICs (0.015 mg/L), indicating imperfect MIC-outcome correlation (*6*).

Itraconazole remains a therapeutic mainstay but might require prolonged high-dose courses, which increases risk for side effects (*7*). Fluconazole and griseofulvin show poorer activity, having higher MICs against *T. indotineae* (*8*–*10*).

A study from Singapore (*11*), this patient’s country of residence, examined whole-genome sequencing of 33 isolates and found nearly 80% of *T. indotineae* isolates were terbinafine-resistant. Azole MICs were generally below European Committee on Antimicrobial Susceptibility Testing wild-type upper limits. No phenotypic azole resistance was observed, and no azole resistance–associated *cyp51A/cyp51B* alterations or gene amplification were detected. Well-known azole resistance mechanisms, including erg11 gene amplification, have been described elsewhere (*12*). This patient’s isolate had a MIC of 0.03 mg/L for itraconazole and 0.5 mg/L for voriconazole, determined later in a research setting using Sensititer YeastOne YO10 plates (Thermo Fisher Scientific, https://www.thermofisher.com) and not available at the time of management. Despite low MICs, clinical failure occurred. MIC for terbinafine was 8 mg/L and determined using Clinical Laboratory Standards Institute M38 methodology (*13*). Echinocandin susceptibility testing was not performed.

Comparable antifungal susceptibility data have been observed across Canada (*14*) and Asia (*15*); terbinafine resistance has been demonstrated to be widespread and MICs for azoles are elevated. The isolates in Canada retained low minimum effective concentrations (MEC) to anidulafungin (MEC_90_ <0.015 mg/L) and micafungin (MEC_90_ <0.015 mg/L) (*10*). In Asia, elevated MICs for azoles were again reported (itraconazole MIC​ 0.5 mg/L, voriconazole MIC 1 mg/L) (*14*). Echinocandins consistently demonstrated low MECs (MEC_90_ <0.004 mg/L for caspofungin, micafungin, anidulafungin). Although MICs and MECs are not directly comparable, those data suggest echinocandins consistently demonstrate strong in vitro activity against *T. indotineae*. However, clinical efficacy of echinocandins remains uncertain; data are limited to in vitro studies (*9*).

Beyond susceptibility data, the previous Singapore study (*11*) also provided epidemiologic insights. Most affected patients were migrant workers, and phylogenetic analysis suggested multiple independent introductions rather than clonal spread. Our patient reported no international travel, raising concern for local acquisition and possible reduced susceptibility that might be emerging beyond that shown in current laboratory data. Enhanced surveillance and access to species-level diagnostics are needed to define transmission patterns and guide public health measures.

In addition, recalcitrant cases should prompt consideration of alternative systemic therapies. Echinocandins have not been routinely used for superficial dermatophytosis and might have limited penetration into keratinized skin (*15*). Prolonged intravenous echinocandin therapy is costly and logistically challenging and carries risk for line-related complications and hepatic enzyme elevation. However, unlike terbinafine and azoles, which target ergosterol synthesis, echinocandins inhibit 1,3-β-D-glucan synthesis, avoiding cross-resistance.

## Conclusions

Given the strong rationale supported by those pharmacological differences and in vitro data, as well as the limited alternative options, combination therapy including an echinocandin was attempted for this patient after multiple standard regimens had failed. Evidence supporting echinocandins for dermatophytosis remains sparse, and this approach should not be interpreted as a standard recommendation. Rather, clinicians should suspect *T. indotineae* when tinea infections fail to respond to first-line therapy and pursue culture with species-level identification where available. Still, this case highlights the potential role of echinocandins for recalcitrant tinea, given the global surge in terbinafine-resistant dermatophytosis and the paucity of effective oral treatment options (*1*,*2*,*7*,*15*).

In summary, although itraconazole remains a first-line treatment for *T. indotineae*, prolonged courses are typically required. Echinocandins might have a role in selected treatment-refractory cases. Further studies are needed to define dosing strategies, tissue penetration, and long-term outcomes to establish the role of echinocandins as antifungal resistance continues to rise. This case highlights therapeutic challenges in Singapore and underscores antifungal stewardship: confirming species-level identification when available, optimizing adherence and conventional therapy, and reserving intravenous agents for truly refractory cases. 
